# Supplementary material for: Complexome profiling of the Chlamydomonas psb28 mutant reveals TEF5 as an early PSII assembly factor
Source: Plant Cell. 2025 Mar 17;37(6):koaf055. doi: 10.1093/plcell/koaf055 (PMC12164590; doi:10.1093/plcell/koaf055)
Supplement: koaf055_Supplementary_Data [file koaf055_supplementary_data.zip › psb28_interactive.zip!/plots/intensity/qualityPlots/peptides/_Cre14.g619350.t1.2.html]

### Cre14.g619350.t1.2

Trivial name:   
SeqCoverage [median]: 16.10  
Mass: 63.64 kDa  
  
 

| Sequence | MissedCleavage | Mass [Da] | wtIntensity | muIntensity | Score |
| --- | --- | --- | --- | --- | --- |
| FKELTAAAPTAPAR | 1 | 1442.79 | 500.23 | 1332.89 | 44.43 |
| GVVLVATSNRPPDDLYK | 1 | 1842.98 | 0.00 | 2079.90 | 51.55 |
| HLEVPLAAGK | 0 | 1033.59 | 0.00 | 845.43 | 91.07 |
| LLVTAAAPPVDLFK | 0 | 1453.85 | 514.70 | 0.00 | 127.42 |
| SAADALPAVR | 0 | 969.52 | 0.00 | 427.00 | 73.26 |
| YEAGLSTGQYRPDPR | 1 | 1708.82 | 0.00 | 1265.33 | 64.65 |
|  |  |  | 1014.9 | 5950.5 |  |
